# Exploring the Barriers of Home Care Services in Iran: A Qualitative Study

**DOI:** 10.1155/2016/2056470

**Published:** 2016-04-05

**Authors:** Heshmatolah Heydari, Hooman Shahsavari, Abdolrahim Hazini, Alireza Nikbakht Nasrabadi

**Affiliations:** ^1^Community Health Nursing Department, School of Nursing and Midwifery, Tehran University of Medical Sciences, Tehran, Iran; ^2^Medical-Surgical Nursing Department, School of Nursing and Midwifery, Tehran University of Medical Sciences, Tehran, Iran; ^3^Firoozgar Hospital, Iran University of Medical Sciences, Tehran, Iran

## Abstract

With increasing chronic diseases, the use of home care is rising in the world. Home care in Iran has many challenges and to improve that, we should identify the challenges and barriers of home care. The aim of this study was to identify and explore the barriers of home care in Iran. This is a qualitative study with content analysis approach that was conducted in Iran in 2015. Fourteen key informants comprising health policymakers, faculty members, nurses, and physicians as well as patients and families engaged in home care purposefully participated in this study. Data was obtained using face-to-face semistructured interviews. A focus group discussion was also used to complete the findings. Graneheim and Lundman's approach was used for analysis of data and Lincoln and Guba's criteria were used to confirm the trustworthiness of study's findings. The data were divided into three main categories and eight subcategories. Main categories included treatment-based approach in the healthcare system, cultural dimensions, and the lack of adequate infrastructure. A position for home care in the healthcare system, considering cultural dimensions in Iranian society and providing an appropriate infrastructure, can be beneficial to improve the situation of home care services in Iran.

## 1. Introduction

The prevalence of chronic diseases is rising in the world [1]; in 2008 over 57 million deaths occurred in the world where 36 million of them were related to the chronic disease. In Iran, 39500 deaths occurred and 76% of them were due to the chronic diseases in 2014 [2]. With considering the important role of community and families in the management of chronic diseases [3], health system in the world has shifted from hospital-based care to community-based care in the last two decades [4].

Home care is a community-based method in the delivery of health services [5], and delivering health services at home can help to reduce the costs, hospital complications, and length of hospitalization [6]. Health systems spend most of their funding on hospitalization and physicians' visit in some countries, that is, about 50% of their total healthcare budget [7]. Many studies showed that home care can decrease the costs as well as promote independence for patients [5]. Patients also prefer to stay in their homes while receiving their healthcare services [8, 9]. Home care services vary from primary to advance services as well as rehabilitation [8] and are delivered differently, in different parts of the world [10, 11]. Because of the numerous benefits of home care such as comprehensive service, control of chronic disease, reducing the costs, minimizing the need for hospitalization, engagement of the community, and increase in patient satisfaction, home care has a special position in today's world [12]. For the effective use of numerous advantages of the home care, identifying barriers and challenges in the home care is essential. In some studies a number of challenges and barriers in home care have been identified in which caring for people with various religions and cultures is one of them [13]. In Markkanen et al.'s study [14], in Massachusetts, home care had many challenges and barriers such as workload, transportation problems, lack of information about family culture, job insecurity, unknown home environment for care providers, low salary, shortage of equipment, and lack of qualified staff among workforce. Genet et al.'s study [10] in Europe shows that home care is different at the international level in regard to general policies, rules and regulations, instructions, payment style, insurance coverage, and target population. Iran is one of the crowded countries in the Middle East [15] that provides health services in general, private, and charity levels. The regulations governing the establishment of counseling centers and nursing care at home in Iran were approved in 2004, but, at the current time, there is no adequate information about situation of agencies that provide home care, quality of home care services provided, and barriers and challenges in the home care [16]. The aim of this study was to identify and explore the barriers of home care services in Iran.

## 2. Methods

This is qualitative research study with conventional content analysis approach.

### 2.1. Settings and Participants

This study was conducted in Iran in 2015. Fourteen individuals including healthcare policymakers, faculty members, nurses, and physicians engaged in the delivery of home care services as well as patients who were receiving home care and their families participated in the study. Participants were selected through purposive sampling. The criteria for participation were to have experience and engagement in the delivery or management of home care for at least one year.

### 2.2. Data Collection

Data were collected by semistructured face-to-face and focus group interviews with the participants [17]. Before each interview, the researcher contacted the participants to set a convenient time, date, and the place for the interviews. To enrich the findings, two face-to-face interviews were held with three of the participants and, in total, 17 interviews were conducted. Moreover, in addition to the individual interviews, one focus group interview was also held to further enrich the findings. Ten people participated in the focus group, six people were from the group who participated in the individual interviews, and four people were new cases. The participants in the study were asked several questions regarding home care in Iran such as how is home care in Iran? What are the barriers of home care in Iran? Moreover, pointed questions, such as please explain more and can you bring an example, were asked to complete the findings. Interviews lasted for 35–45 minutes and the length of the focus group meeting was 40 minutes. The sampling was continued until reaching data saturation and obtaining no new data from the interviews [18]. All of the interviews were recorded by digital device. Collection and analysis of data were done simultaneously.

### 2.3. Analysis

Graneheim and Lundman's content analysis approach was used for analysis of data [19]. Therefore, in this study, after each interview, a transcript of the interview was written and studied several times in order to extract the primary codes. The primary codes that were similar in terms of the meaning and concept were sorted in categories according to their similarities. Finally, latent meanings emerged from the data.

### 2.4. Trustworthiness

Lincoln and Guba's criteria of credibility, dependability, and conformability were used to confirm the trustworthiness of study's findings [20]. Researchers tried to have prolonged relationship with participants in order to gain their trust which could help to obtain honest information from them. Emerging primary codes from the interviews were returned to the participants in order to determine whether our findings confirm their viewpoints and if the codes were inconsistent with their viewpoints, they were corrected. Two faculty members specializing in qualitative research also helped to approve the final codes. Then, the approved codes were divided into main categories and subcategories. Moreover, in this study an attempt has been made to find suitable participants who had a maximum variation of age, gender, working position, expertise, and work experience.

### 2.5. Ethical Considerations

Ethics Committee of Tehran University of Medical Sciences, Tehran, Iran, has also approved this study. Code of ethical approval is IR.TUMS.REC.1394.175. All of the participants were informed about the aim and method of the study. Moreover, they were informed that participation in the study is voluntary and they can leave the study at any time. They were assured about confidentiality of their information and management of data anonymously. Finally, written informed consent was obtained from all participants.

## 3. Results

Nine males and five females with the mean age of 41 ± 7.1 and the mean work experience of 18 ± 3.6 years participated in the study. In this study, 927 codes were generated which were divided into three main categories and eight subcategories (Table 1). Main categories and subcategories are explained as follows (Table 1).

### 3.1. Treatment-Based Approach in Health System

The first main category of the study was treatment-based approach. Health system in Iran has a treatment-based approach and prevention comes second. Therefore, the priority of health system is to provide and increase the number of beds in hospitals. Community-based care and home care do not have any position in Iran's health system. This main category consists of three subcategories including one-dimensional management in the health system, prioritization of hospital care over community-based care, and emphasis on the treatment rather than prevention in the education system.

#### 3.1.1. One-Dimensional Management in the Health System

According to our participants, one-dimensional management in the health system is a barrier in home care. Most of the officials in Iran's health system are specialized physicians and they think home care is a threat to their income.

*…managers in the health system are physicians and they think this program is in conflict with their interests…(p 5). …Because of the doctor dominant health system, policymakers have a negative attitude towards home care…(p 5). …at the current time, one percent of specialists refer their patient to me for home care services…. (p 5)*

#### 3.1.2. Prioritization of Hospital Services over the Community Services

The study findings highlighted the increase of treatment centers in Iran's health system. Most of the nurses and physicians employed in the treatment centers are practicing in the secondary levels of prevention. Home care does not have any position in Iran's health system.

*…Health system of Iran waits for people to get sick and then treat them…(p 4). …prevention at primary level is excellent, but we have to leave the primary level because of urgency in the secondary level…. (p 4)*

#### 3.1.3. Defect in the Education System

According to our participants, education system is not efficient in training healthcare professionals capable of delivering home care services. Medical and nursing graduates are unable to provide off-hospital services.

*…nurses working in hospital are unable to provide home care services independently…(p 1). …nurses in our colleges get trained for bedside care, whereas home care service is different…. (p 2) *

### 3.2. Cultural Dimensions

Culture is the sum of behaviors, beliefs, values, and symbols of a group of people. People's culture is considered as one of the most effective elements in home care. Home care takes effect from lifestyle, attitude, beliefs, and viewpoint of the community. This main category consists of two subcategories including distrust of community towards nonphysician experts and defect in the safety of healthcare providers and families.

#### 3.2.1. Distrust of Community towards Nonphysician Experts

According to our participants, negative viewpoint of the community and their distrust towards nurses are considered as one of the barriers in home care delivery.

*…our people' perception of a nurse is someone who inject ampules and that is all…(p 2). …why position of nurses is so low in my country? People have more trust towards nurses in hospitals (because of the presence of physicians) than nurses who work alone in the community…(p 2). …families also prefer to receive care from physicians…. (p 1)*

#### 3.2.2. Defect in the Safety of Care Providers and Families

According to our participants, one of the barriers in home care delivery was the lack of safety and security for care providers and families. Participants expressed that the care providers enter their personal space without predetermined planning.

*…nurses that entered into patients' homes for care delivery, were worried about an assault to their privacy…who is responsible about this problem?…. (p 1) *

### 3.3. Infrastructure Problems

Some factors are necessary for achieving goals and success and survival of a program depends on them. Interpretation of data showed that, for the success of home care service, insurance coverage, execution of protocols, and interprofessional and intersectional cooperation have important roles. This main category consists of three subcategories including lack of insurance coverage, absence of clear executive protocols, and defect in the interdisciplinary cooperation.

#### 3.3.1. Lack of Insurance Coverage

According to our participants, home care was an expensive service and was not covered by public insurance. Most of the participants expressed that insurance coverage has a key role in the success of home care program.

*…health insurance is a civil right for people, but does not cover home care services in Iran…(p 2). …if insurance cover some of the costs, people don't shift towards the informal agents…. (p 5)*

#### 3.3.2. Absence of Executive Protocol

Interpretation of data showed that there is no clear instruction for assessment, categorization of patients, payments, salaries, estimate of costs, and determining the competency and qualification of care providers in the home care system. Participants expressed that protocols can help to solve the problems between nurses and home care centers and promote the quality of services and patients satisfaction.

*…It isn't any framework for us…about costs, incomes, cares…(p 7). …we categorize patients ourselves, and don't have any instruction for that…. (p 6) *

#### 3.3.3. Defect in the Interdisciplinary Cooperation

According to our participants, providing and promoting healthcare is a responsibility of all social organizations. Yet, Iran's healthcare system is unable to provide that. They also expressed that interprofessional and intersectional cooperation are essential in home care.

*…nurses delivering home care need to counsel a physician…(p 3). …Municipal and security police can facilitate home care…. (p 12) *

## 4. Discussion

Study's findings revealed that factors such as treatment-based health system, cultural factors, and absence of infrastructure are the most important barriers in the delivery of an effective home care in Iran. The findings of this study showed that healthcare system in Iran has the physician-based management; healthcare system reform is necessary [21]. According to WHO, physician-based management is considered as one of the threats in the health system of Iran [22]. Findings also showed that, in the health system of Iran, hospital-based services are more preferred than community-based services which have no position in Iran's healthcare system. While, with the change in the demographic characteristics, authorities in the world are forced to use community-based care [23], most of the European countries have considered home care as a priority in their health system [10] and have decentralized home care management and policy [21, 24]. In Iran, high statistics of chronic diseases [2] are in favor of necessary reform in the health system [21, 25, 26]. Changing the health system from treatment-based to community-based and the use of successful models and experiences of other countries can help to improve home care in Iran.

One of the barriers in home care was the lack of qualified staff capable of delivering home care services. In Iran the purpose of curriculum in nursing schools is to train nurses with community viewpoint [27], but the graduates in this field do not have sufficient professional attitude and skills to provide services in the community [28], whereas, in the developed countries, volunteer nurses for home care services must be trained for 3 to 4 years and should be recertified during their work [29]. In Denmark nurses graduating from college after 3 to 5 years of training are able to perform tasks such as assessment, planning, prevention, and treatment of all patients [29]. But in Iran the majority of nursing schools train their students for hospital-based services [28]. With a change in the trend of diseases [2] and a move from hospital- to community-based care [30], it is necessary that curriculum of healthcare education should also change in Iran.

The findings also showed that cultural dimensions were one of the barriers in Iran's home care. Cultural qualification is considered as one of the pillars in the nursing care and care providers should consider every patient as a unique individual [31]. Many studies show that if care providers become familiar with the language and tradition of the clients, cooperation between them is better [32]. On the other hand, cultural heterogeneity between provider and client can cause care provider to pay more attention towards physical dimension and psychological dimension of clients is neglected [33]. However, very few researches have been conducted regarding the cultural competency in the world [34]. Inadequate awareness about the cultural needs of the people is a factor for misunderstanding in the interaction between care provider and clients [35]. Providers should gain adequate knowledge about the culture of clients for a better interaction [36, 37]. In this study, one of the cultural factors was distrust to nonphysician staff. Similarly, findings of the study showed that people were more interested to receive service from physicians [34, 38], while nonphysician staff were also capable of providing the same services for patients [39]. According to WHO if a health system wants to meet the health needs of the community, it has to use nurses and midwives [40]. Therefore, it is necessary to explain to the community the important role of the nurses in providing healthcare services at home.

The findings of this study indicated that one of the barriers in home care is the lack of adequate safety and security for care providers and families. Violence and instability of patients and their families were also one of the challenges in the home care [41]. Home care is delivered in an environment that physical and mental hazards exist [14] and providers may even be at risk of other hazards such as needle stick injury and contagious diseases [42]. Therefore, it is necessary that health authorities look for ways to solve these issues.

Findings in this study showed that another barrier in home care was the absence of an appropriate infrastructure. WHO emphasizes that public should be covered by universal insurance and even uses the universal health coverage (UHC) term for that [21]. According to some studies, home care services are covered by insurance in the majority of developed countries [10], whereas WHO's report indicated that Iranians paid more than 88% of their healthcare costs from their own pocket in 2013 [10, 43]. Thus, any decrease in the healthcare costs can encourage people to use home care services. Yet, the lack of insurance coverage for home care services has caused people to seek home care from unofficial sources [43]. Coverage of home care services by insurance can lead to a regulated home care and encourages people to use home care services.

Findings in this study revealed that one of the important barriers of home care was the absence of executive instruction in the recruitment of healthcare staff, disproportionality between care provider and client, and lack of clear estimate of the services' costs. Some of the developed countries have specific protocols for their home care services [44–47], and clients' needs and service costs are assessed by these protocols [48]. Some other countries have national guidelines for informal providers to deliver elementary services [48]. Therefore, providing appropriate instructions and guidelines can help to improve Iran's home care services.

Findings in this study showed that another barrier of home care services was ineffective interdisciplinary cooperation. According to WHO's report, one of the threats for health systems is weakness in the intersectional cooperation [5], while interprofessional cooperation in the home care helps to promote quality of life, social and physical activities [49], and safety of clients [50]. Majority of developed countries are using the capacities of municipalities for home care services [10]. Cooperation and coordination between hospitals and community healthcare teams can also lead to a reduction of readmission and promote the quality of services. This program is used in the majority of European countries; for example, in Finland, municipalities in every region can have access to patients' record electronically in order to facilitate continuation of their care at home [29]. Therefore, reinforcement of interprofessional and intersectional cooperation such as cooperation between health centers and municipalities can help to promote healthcare services.

One of the limitations in this study was the lack of information about home care in Iran's healthcare system. Even policymakers and healthcare professionals engaged in the delivery of home care were unfamiliar with the dimensions of home care in Iran. This study has been conducted by qualitative method; therefore, the findings of this study do not have the ability to be generalized to other communities. More researches in this field are recommended to reveal all dimensions of home care in Iran.

## 5. Conclusion

This study concludes that a position for home care in Iran's healthcare system, considering cultural dimensions in the Iranian society and providing an appropriate infrastructure such as insurance coverage, designing an executive instruction, and reinforcement of interdisciplinary cooperation, can be helpful for the improvement of home care in Iran.

## Figures and Tables

**Table 1:** Barriers of home care in Iran.

Treatment-based approach in health system	One-dimensional management in health system
	Priority of hospital services over community services
	Defect in the education system

Cultural dimensions	Community distrust to nonphysician experts
	Defect in the safety of care providers and families

Infrastructure problems	Lack of insurance coverage
	Absence of executive protocols
	Defect in the interdisciplinary cooperation
